# Modelling the mechanical properties of concrete produced with polycarbonate waste ash by machine learning

**DOI:** 10.1038/s41598-024-62412-5

**Published:** 2024-05-21

**Authors:** S. Sathvik, Rakesh Kumar, Nestor Ulloa, Pshtiwan Shakor, M. S. Ujwal, Kennedy Onyelowe, G. Shiva Kumar, Mary Subaja Christo

**Affiliations:** 1 Department of Civil Engineering, Dayananda Sagar College of Engineering, Bengaluru, Karnataka 560111 India; 2 Department of Civil Engineering, National Institute of Technology, Patna, India; 3 Facultad de Mecanica, Escuela Superior Politecnica de Chimborazo (ESPOCH), Panamericana Sur km. 1 ½, 060155 Riobamba, Ecuador; 4 Technical College of Engineering, Sulaimani Polytechnic University, Sulaymaniyah, 46001 Iraq; 5 Department of Civil Engineering, Michael Okpara University of Agriculture, Umudike, Nigeria; 6 Department of Civil Engineering, Kampala International University, Kampala, Uganda; 7 Department of Networking and Communications, School of Computing, SRM Institute of Science & Technology, Kattakulathur, Tamil Nadu 603 203 India

**Keywords:** Polycarbonate waste, Mechanical properties, Durability properties, Microstructural study, Machine learning, Cost analysis, Engineering, Materials science, Mathematics and computing

## Abstract

India’s cement industry is the second largest in the world, generating 6.9% of the global cement output. Polycarbonate waste ash is a major problem in India and around the globe. Approximately 370,000 tons of scientific waste are generated annually from fitness care facilities in India. Polycarbonate waste helps reduce the environmental burden associated with disposal and decreases the need for new raw materials. The primary variable in this study is the quantity of polycarbonate waste ash (5, 10, 15, 20 and 25% of the weight of cement), partial replacement of cement, water-cement ratio and aggregates. The mechanical properties, such as compressive strength, split tensile strength and flexural test results, of the mixtures with the polycarbonate waste ash were superior at 7, 14 and 28 days compared to those of the control mix. The water absorption rate is less than that of standard concrete. Compared with those of conventional concrete, polycarbonate waste concrete mixtures undergo minimal weight loss under acid curing conditions. Polycarbonate waste is utilized in the construction industry to reduce pollution and improve the economy. This study further simulated the strength characteristics of concrete made with waste polycarbonate ash using least absolute shrinkage and selection operator regression and decision trees. Cement, polycarbonate waste, slump, water absorption, and the ratio of water to cement were the main components that were considered input variables. The suggested decision tree model was successful with unparalleled predictive accuracy across important metrics. Its outstanding predictive ability for split tensile strength (R^2^ = 0.879403), flexural strength (R^2^ = 0.91197), and compressive strength (R^2^ = 0.853683) confirmed that this method was the preferred choice for these strength predictions.

## Introduction

Concrete is the most widely used building material in the world, so it needs a large amount of cement and aggregates^1^. The cement industry and aggregate quarries need to be operated longer to accommodate the increased demand for these components. Both operations cause major environmental problems^2,3^. However, healthcare institutions contribute to environmental and waste management issues by producing waste. Poly-carbonate waste is produced in healthcare services^4,7^. Different regions have varying amounts of trash. The presence of dangerous materials means that special care must be taken when disposing of this trash^8^. Although many countries have developed laws and regulations to address waste, the quantity of waste produced and the implementation of regulations differ from place to place^9,10^. The majority of this trash is nonhazardous, and it is possible, with appropriate treatment, to transform it into a product that may be reused^7,9,11,12^.

The amount of biological waste produced daily per bed will differ from one hospital to another based on the nature of the illnesses, the nature of the care provided and the hospital waste management policies^13–15^. It ranges from 1 to 2 kg in developing and underdeveloped countries to 4 to 5 kg in advanced countries. In developed countries, 0–15% of the waste is infectious; however, in India, it ranges from 45.5 to 50% and needs specific treatment^10,16,17^. Polycarbonate waste generated from hospitals, medical research activities and other medical sources is a major problem in India^18–21^. Polycarbonate waste is distinct from any waste created during the diagnosis, treatment, or immunization of humans or animals; during related examination activities; or during the production or experimentation of biologicals^22^.

There is an increasing need for effective waste management strategies as a result of the accumulation of plastic waste^5,23^. The use of polycarbonate in products such as electronic devices and automotive components makes it stand out from other waste plastic types^13,21,24^. Through controlled thermal treatment, it is possible to convert polycarbonate waste into ash, which can be used for its sustainable disposal^16,25^. As a supplementary material, it has yet to be extensively explored as a concrete additive. By examining the performance of concrete with different fractions of PC waste ash, this study aims to fill this knowledge gap^6^.

Polycarbonate waste is dangerous because it has inherent potential for the spread of infection, both acquired infections within health-care facilities and the risk of infection to people working outside of health care facilities, such as waste collectors, scrounging staff, and the common people. According to statistics, sharps injuries account for 60% of all injuries reported by hospital employees^17^. The pollution produced has negative effects on both the natural world and the human world. Another difficulty in developing nations such as India is the lack of available landfills for trash disposal^5,6,16,17^. The availability of chemicals and harmful compounds in waste creates a new problem of land contamination, as trash is typically dumped far from urban areas, often close to or even on farming land^7^. Therefore, it is essential to dispose of waste in accordance with applicable national and international laws and regulations^4^.

According to a study conducted in Pune, India, PC waste could have a number of consequences, and responsible waste management is essential^10^. Research has shown that polycarbonate waste ash can be used as a supplementary cementitious material. Incineration of the garbage results in ash. However, such materials can be used to replace some of the cement in fresh concrete^26^. Despite variations in waste quantity, this action will not only help with the problem of managing PC waste but also provide a material that can be used for partial replacement of cement in buildings^23^. There has been research on the possibility of using polycarbonate waste to create ash for use in concrete. Workability is a major consideration among other factors when determining the quantity of ash produced from PC waste, along with fineness and the water-cement ratio^25^.

According to the literature, adding fly ash to concrete decreases its workability^27^. The chemical and physical properties of waste are within the limits established by regulations for used quarry dust. Compared with the fresh specimens, the 90-day-cured specimens show a 25–54% reduction in water absorption. This study details the findings of an experiment conducted to examine the impact of partially replacing cement with PC waste ash^16^. This study examined the workability and density of fresh concrete, as well as the compressive strength of hardened concrete after 7 and 28 days^28^. However, these wastes can be successfully utilized in the manufacturing of concrete, thereby decreasing the demand for land to dispose of PC waste ash and protecting the environment by reducing the consumption and production of cement^6^

The potential toxicity and harm caused by PC waste typically depend on where it is derived from. The strongly poisonous metals it contains are extremely dangerous to human health. Presently, there are 170 general polycarbonate waste treatment facilities with a total of 140 incinerators distributed across the country^29^. In concrete, waste from medical practices can be utilized as a weight-for-weight replacement for cement. Ashes from medical facilities can be mixed with cement to create a material that can be utilized for building construction^30–32^. Additionally, it can be utilized in the construction industry as a stabilizing agent in asphalt and road pavements.

Most previous studies have examined lower replacement percentages, approximately 20% or less by weight. Thus, a key novelty of this work is the testing of increased polycarbonate waste ash percentages, up to 25% for partial cement replacement, to analyse the effects on key properties such as workability, compressive strength, flexural strength, acid resistance and water absorption^10^. Evaluating concrete performance at higher polycarbonate waste ash contents can help determine optimal usage amounts to balance material performance with the sustainability benefits of utilizing this waste material^33^. This is important because it can help mitigate environmental issues associated with cement production and polycarbonate waste disposal. Successfully utilizing polycarbonate waste ash in concrete can also lead to reduced reliance on cement, lowering the carbon footprint of concrete^34–36^.

Conventional techniques for estimating concrete strength typically rely on linear and nonlinear regression analysis. To fully understand the performance of waste-based concrete, extensive testing is necessary due to its diverse behavior. Despite this, the process is costly, time-consuming, and requires many ingredients. Therefore, due to their computational and knowledge processing capabilities, machine learning (ML) techniques such as artificial neural networks (ANNs), fuzzy logic (FL), adaptive neuro-fuzzy inference systems (ANFISs), and genetic algorithms (GAs) can be used as good substitutes for extensive testing to predict the various characteristics of concrete. These techniques are essentially computer algorithms that improve automatically based on prior experience and measured data. Due to its ability to analyse high-dimensional variables and address variable collinearity, ML has found widespread use in addressing the limitations of traditional methods for predicting concrete performance^37–40^. Young et al.^41^ proposed a method for predicting the performance of industrial concrete. To forecast the shear and compressive strengths of concrete, Chaabene et al.^42^ investigated the practicability of employing a number of algorithms, such as artificial neural networks, decision trees, support vector machines, and evolutionary algorithms. Li et al.^43^ conducted a thorough assessment of the current state of ML models and their applications to concrete performance metrics (such as slump, splitting compressive strength, compressive strength, and compressive strength). Predicting desired properties in waste aggregate-based concrete will therefore benefit from the application of ML techniques. To achieve the required qualities at the lowest possible cost, it is also vital to optimize the various components used to make concrete.

The novelty of this study is that research on PC waste has not been widely carried out for the replacement of cement with PC waste ash in concrete, and the mechanical and durability properties of the waste waste have not been determined. The machine learning models applied were the least absolute shrinkage and selection operator (LASSO) regression (LR) and decision tree (DT) algorithms. This study presents new methods for forecasting concrete properties that incorporate PC waste. These algorithms for polycarbonate waste concrete have not been the subject of any prior research, even though several ML approaches have been investigated previously. DTs provide interpretability and simplicity, which makes them appropriate for complex datasets, while LRs help with feature selection and multicollinearity. By addressing this gap in the literature, this research offers useful guidance for optimizing concrete mix designs that incorporate PC waste.

This study consists of six main sections. The second section defines the various mixtures used to combine PC waste ash as a substitute for cement in concrete, specifically with the mix proportions of M25-grade concrete. The third section describes the details of the experimental tests conducted on concrete replaced with PC waste ash. The fourth section compares the results of numerous experiments between traditional concrete and concrete mixed with polycarbonate waste ash. The fifth section describes the water absorption and acid resistance tests between conventional concrete and polycarbonate waste ash concrete. Finally, strength- and microstructural-related investigational studies are addressed in the sixth section.

## Materials and methods

### Methodology

**Figure 1:**
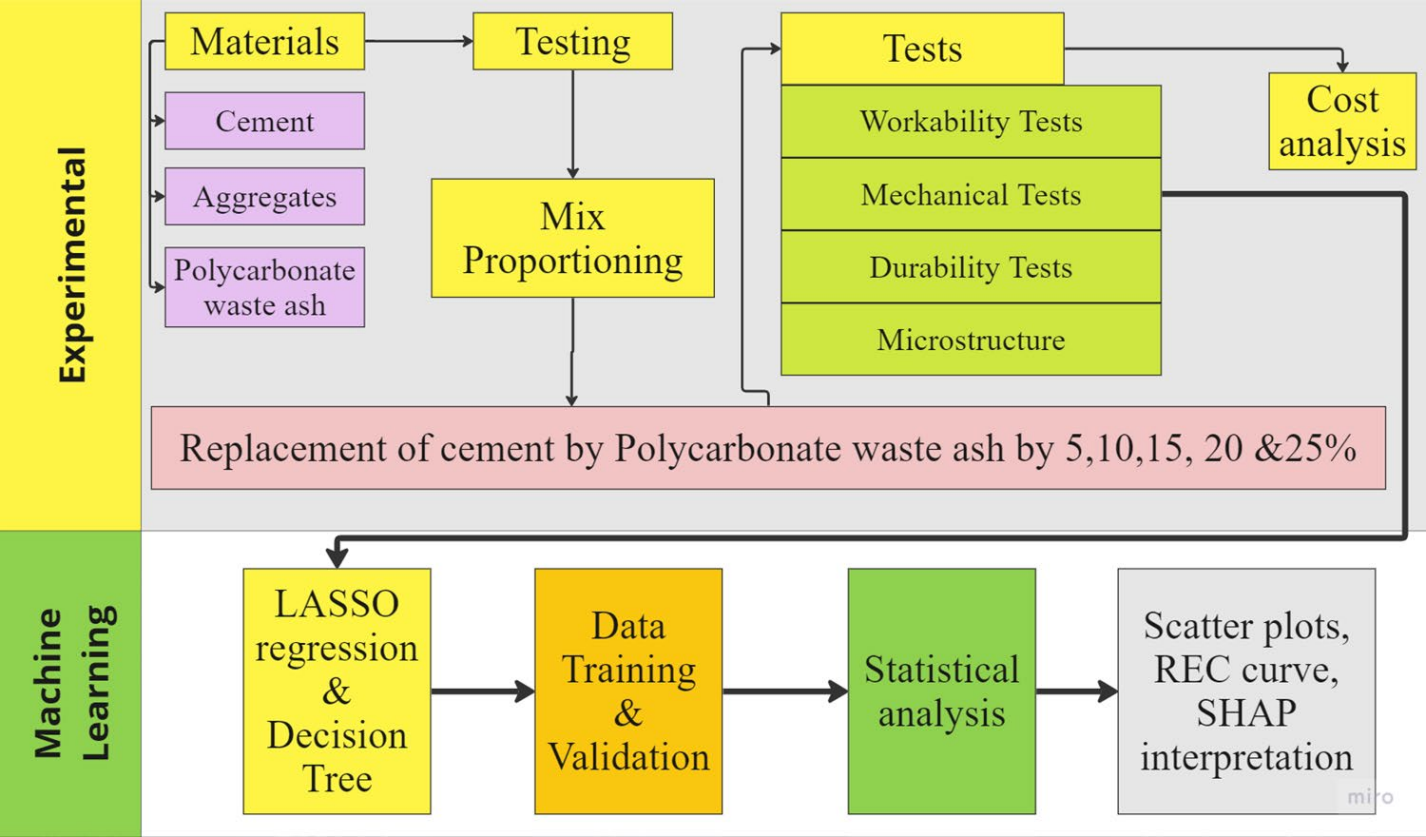
Flow chart of the methodology.

The methodology shown in Fig. 1 involves conducting experimental material testing for concrete production, including workability, mechanical, and durability tests, along with incorporating polycarbonate waste ash as a partial replacement for cement. Additionally, machine learning techniques such as least absolute shrinkage and selection operator (LASSO) regression and decision tree analysis were applied for data analysis and prediction. The process includes material characterization, mix proportioning, cost analysis, and statistical analysis of the results.

### Materials

**Figure 2:**
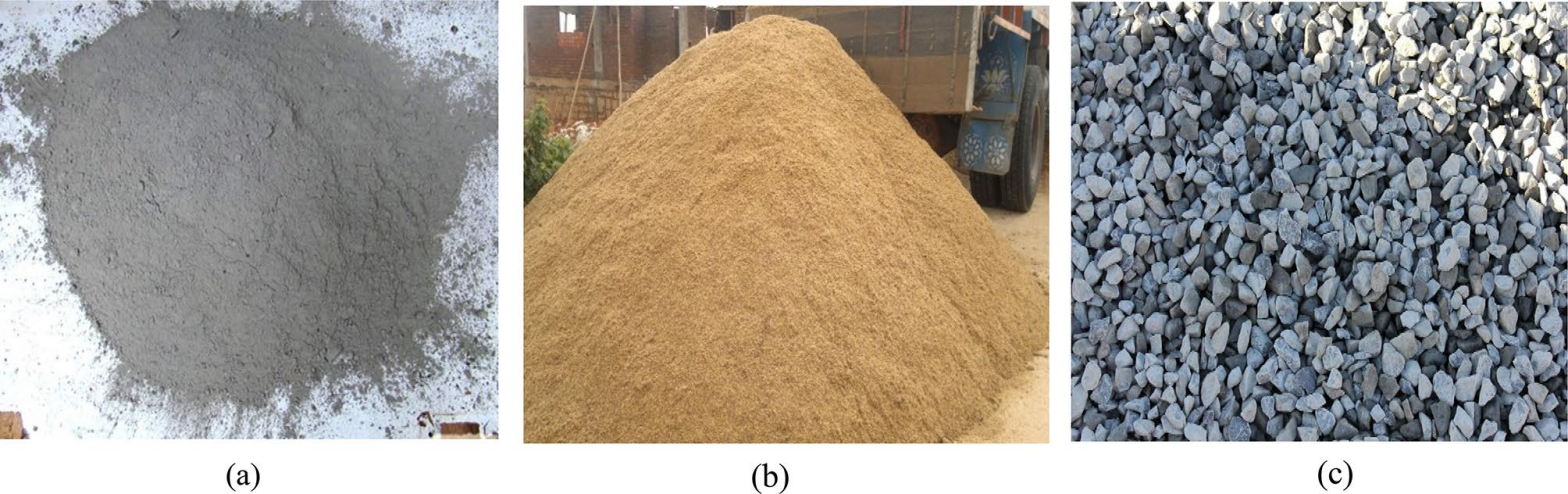
(**a**) Cement (**b**) Natural sand (**c**) Crushed stone aggregates.

In the current study, a research study and experimental program were carried out to examine the suitability of using polycarbonate waste ash as a fractional replacement of cement in concrete, as well as the effect of replacing cement with polycarbonate waste ash on the workability of concrete in general and on the compressive strength in particular^25^. This research used 53-grade ordinary Portland cement that conforms to the IS 12269-2013 standards. It has been shown that cement has a specific gravity of 3.16. The proportions of the mix that was utilized for conventional concrete were 1:1.96:3.03:0.45, and the cement content was 390 kg/m^3^, as shown for all the materials in Fig. 2.

The fine aggregates employed in this study were composed of pure river sand that passed through a 4.75 mm sieve with no oversized particles, confirming the presence of gradation zone III. The fine aggregate was natural river sand, which complied with the requirements of the Indian Standard IS 383: 2016. Most of the particles in fine aggregates are smaller than 9.75 mm in size; hence, they are typically made up of natural sand. A common type of fine aggregate, natural sand, which is cleaned and sieved to extract particles larger than 5 mm, is used in construction. Particles with a diameter of more than 4.75 mm but typically between 9.75 and 37.5 mm are considered coarse aggregates. Mining coarse aggregate from rock quarries or dredging it from river channels means that its size, shape, hardness, texture, and many other attributes can vary substantially depending on where it was extracted. Coarse aggregates are often characterized by their relative density (or specific gravity), bulk density, and absorption, all of which are important in describing their behavior.

Workability, compressive strength, permeability and watertightness, durability and weathering, drying shrinkage, and possible cracking are some of the fresh and hardened qualities of concrete that are controlled by the amount of water in the concrete. Cement composites require portable water for mixing and curing prior to use. Water has a pH of 6.5 and conforms to all other quality criteria established by Indian standards.

#### Polycarbonate waste ash

**Figure 3:**
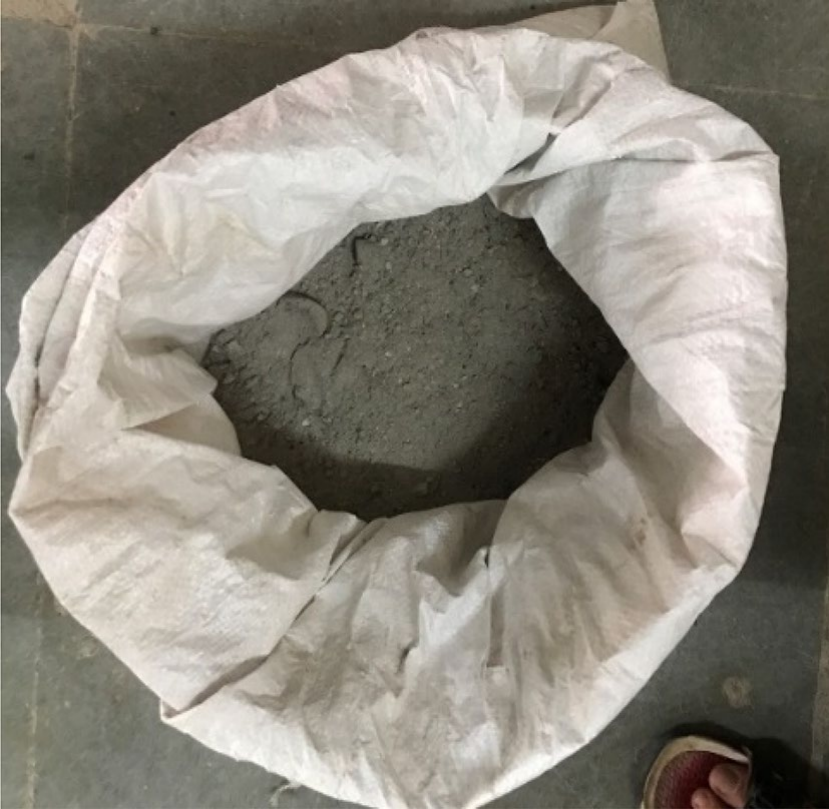
Polycarbonate waste ash.

The polycarbonate waste ash utilized in this study was acquired from Eco Industry Private Limited in Bangalore, as shown in Fig. 3. Polycarbonate wastes include microbiological and biotechnological wastes such as hypodermic needles, syringes, scalpels, and broken glass. The ash from PC waste collection can be both primary waste and secondary waste. For M25, the maximum allowable water-to-cement ratio was calculated to be 0.45 under moderate exposure conditions.

#### Material specification

The materials must have enough data on their specific gravity and water absorption, which can affect the mechanical behavior of the structure. Therefore, Table 1 shows the main material properties of the cement and the fine and coarse aggregates.

**Table 1:** Physical properties of the polycarbonate waste ashes.

Sl. no	Physical constituents	
1	Specific gravity	3.16
2	Loose bulk density(kg/m^3^)	3160
3	Loss on ignition (%)	1.1
4	Specific surface(m^2^/g)	2.35
5	Soundness (mm)	8.2

### Mix-propulsion

By using 0%, 5%, 10%, 15%, 20% and 25% PC waste ash as fractional substitutes for cement in concrete, the purpose of this research was to analyse and assess the qualities of fresh, physical, and hardened concrete, as shown in Table 2. This was accomplished by using biomedical waste ash as a partial substitute for cement in concrete. Sixty different shapes of concrete were cast, including cubes, cylinders, and prisms. The mix ratio was M25, and the water-to-cement ratio (w/c) was 0.45. Following the casting process, each specimen was placed in a curing tank for 7 or 28 days before being evaluated using a universal testing machine (UTM)^25^. In accordance with the procedure outlined in the ASTM C 192 code, the specimens were cast in the form of standard cubes measuring 100 mm on one side, cylinders measuring 100 mm in diameter and 200 mm in height, and prisms measuring 100 mm on one side and measuring 500 mm in length. The compressive, split tensile, and flexural strengths of this concrete were determined using the specimens. Moreover, the density and water absorption of the concrete specimens were examined after 28 days. For each ratio, three different concrete specimens were cast, and the final result was averaged to determine the effectiveness^16^. Scanning electron microscopy (SEM) and energy dispersive X-ray (EDX) analysis were conducted on the concrete samples to characterize the microstructure and chemical composition. A cost analysis was performed by calculating and comparing the costs of raw materials for conventional concrete and 20% polycarbonate waste ash concrete mixes. The market rates for cement, aggregates and waste ash were used for the estimation^6^.

**Table 2:** Concrete mixes prepared with the control and different percentages of ash.

Sl. no	Material replacement	Cement %	Polycarbonate waste %	Fine and coarse aggregate %	Mix ratio	W/C ratio
1	0% Polycarbonate waste ash	100	0	100	1:1.96:3.03	0.45
2	5% Polycarbonate waste ash	95	5	100	1:1.96:3.03	0.45
3	10% Polycarbonate waste ash	90	10	100	1:1.96:3.03	0.45
4	15% Polycarbonate waste ash	85	15	100	1:1.96:3.03	0.45
5	20% Polycarbonate waste ash	80	20	100	1:1.96:3.03	0.45
6	25% Polycarbonate waste ash	75	25	100	1:1.96:3.03	0.45

### Testing methods

The concrete casting, curing, and testing were all performed as per the codal provisions provided by the Indian standards and ASTM standards. These standardized tests allow for reproducible evaluation of fundamental concrete properties that are indicators of performance and durability. The workability test was carried out via the slump cone method (IS 1199), which provides insight into the effect of the mix proportion on the workability of concrete. Mechanical properties such as compressive strength, as determined by IS 516, determine the main strength property that governs concrete design, and split tensile tests on cylinders (IS 5816) and flexural tests on prisms (IS 516) provide supplementary strength criteria. Furthermore, durability factors such as water absorption (IS 1199) and acid resistance, which influence long-term performance, were examined. These test procedures have been selected and standardized for their reliability in assessing the basic properties of concrete containing supplementary materials. Using widely accepted test methods ensures that the results can be compared to the literature benchmarks and recommendations.

#### Workability test

This experimental study employs a slump test, which is the most common method for measuring the consistency of concrete. The examination was conducted according to IS 1199–1917. Workability is a property of fresh concrete that is determined by the amount of internal work required to achieve full compaction of the concrete without bleeding or segregation.

#### Compression test

The compression test was conducted in accordance with IS: 516, 1959, to determine the material’s strength. To cast sixteen mixture proportions, 150 mm × 150 mm × 150 mm molds were used. The test was performed after 7 and 28 days of curing using a 3000 kN capacity universal testing machine^37,38^.

#### Split tensile strength

The specimens for the test were cast using cylindrical molds 150 mm in diameter and 300 mm in length. The load is applied to the universal testing machine to determine the compliant splitting tensile strength IS 5816-(2004). Twelve cylinders are cast for both the control mix and the sustainable concrete mix. At 7 and 28 days of curing, tests were conducted to compare the observations.

#### Flexural strength

The flexural test is performed based on the IS 516 (1959) and cast prisms to determine the flexural load carrying capacity of the specimens using a universal testing machine with a 20-ton capacity. Twelve prisms were cast for the control mix, twelve prisms were cast for the optimal mix, the results were compared, and variations were noted.

#### Durability tests

The water absorption test was conducted as per IS 1199–1959 on 100 mm concrete cubes cured for 28 days. The dried samples were immersed in water for 24 h, and the increase in weight was used to determine water absorption. For the acid attack test, 150 mm cubes were cast and cured for 28 days.

The Indian Standard (IS) code for testing acid attack on concrete is IS 456:2000, which outlines procedures for assessing concrete resistance to various chemical attacks, including acids. The process involved exposing concrete cubes or cylinders to 5% H2SO4 solution for 60 days in acid solutions of specified concentrations and durations, and weight loss was used to quantify acid resistance.

## Machine learning methodology

### LASSO regression (LR)

By encouraging sparsity in the feature set, the LASSO regression (LR) algorithm is unique in the field of regularized linear regression in that it can reduce overfitting^46^. Equation (1) shows the optimization objective function at the center of the LR. The goal in this case is to limit the sum of the absolute values of the regression coefficients and minimize the sum of the squared residuals. The fundamentals of LASSO regression are encapsulated in Eq. (1), wherein every term is crucial in determining the behavior of the model. The goal of the first term is to minimize the difference between the values that were predicted and those that were observed, which is the standard linear regression objective. In contrast, a regularization term is introduced in the second term, and its magnitude is determined by the hyperparameter α. Here, m is the number of samples, ${\theta }_{i}$ is the regression coefficient related to the ${i}_{th}$ input variable, ${x}_{i}$ is the ${i}_{th}$ input variable, $b$ is the bias term, and ${y}_{i}$ is the ${i}_{th}$ sample’s actual value. LASSO regression provides a compelling framework for reliable and understandable model building by carefully balancing the objectives of penalizing excessive complexity and fitting the data.

1 $$J\left( \theta \right) = \frac{1}{2}\mathop \sum \limits_{i}^{m} \left( {\theta_{i}^{T} x_{i} + b - y_{i} } \right)^{2} + \alpha \mathop \sum \limits_{j}^{n} \left| {\theta_{j} } \right|$$

**Figure 4:**
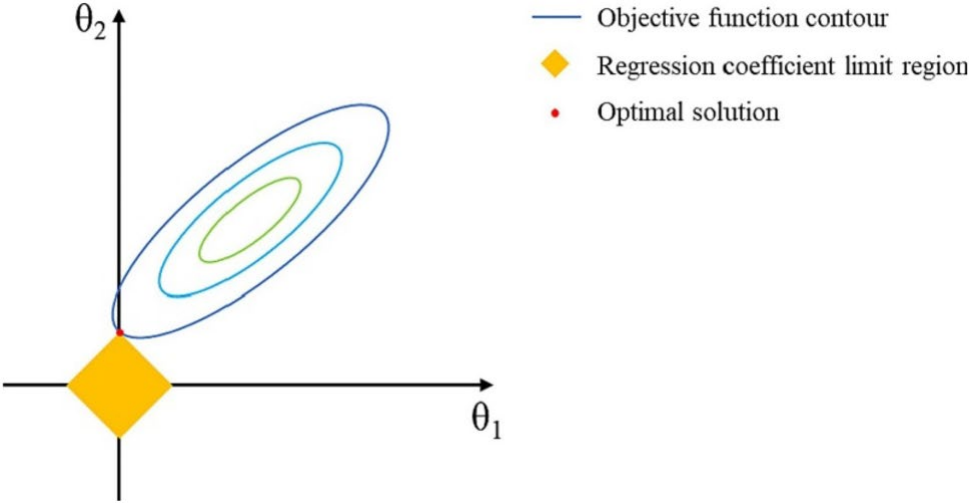
Schematic diagram of the LR algorithm.

A graphic representation of the LR algorithm is shown in Fig. 4, which also offers a clear understanding of its fundamental ideas. The contour of the objective function in the schematic representation is represented by an ellipse, which functions as a visual aid for the optimization procedure. Simultaneously, the yellow diamonds represent the boundary region of the regression coefficient and the limitations imposed by regularization. The search for the ideal solution, symbolized by the red tangent point, is the fundamental component of the LR algorithm. This point, which is positioned between the yellow diamond and the ellipse, represents a perfect balance—a fine balance at which the model attains both limited complexity and low error. It is noteworthy that this equilibrium point appears with a regression coefficient value of zero, indicating a level of sparsity that is desirable. Above all, by varying the hyperparameter $\alpha$, the LR algorithm gives practitioners the ability to manoeuvre through this complex environment. By carefully adjusting α, LR efficiently reduces the parameter space, reducing the possibility of overfitting while maintaining model performance. Regression analysis benefits from the interpretability and robustness that LR provides, making it a flexible tool.

### Decision tree (DT)

A key component of supervised learning, the decision tree (DT) provides a flexible method for addressing problems related to both regression and classification^38,40,41^. Its hierarchical structure contains complex classifications, with each node denoting a crucial dataset attribute. The use of regression techniques seamlessly integrating DTs to extrapolate predictions based on independent variables is needed^38,42,43^. To put it simply, the DT is a tree in its purest form, with its nodes acting as decision points that direct the path toward definitive conclusions^42^. The root node is at the center, and it branches out to encompass a plethora of options, much like the widely spaced branches of a real tree. With several branches, every decision node has the authority to choose what to do, and the leaf nodes gracefully complete as the final results of the decision-making process^18,45,46^. The decision tree’s methodical approach to data analysis and elegant structure are what make it so appealing. The algorithm carefully assesses the differences between the actual and predicted values by wisely dividing the data samples at key points. Errors are carefully examined at each bifurcation, leading to fitness function-based optimal split point selection. With each iteration resulting in increased clarity and predictive accuracy, this iterative process guarantees the decision tree model’s evolution and improvement.

## Descriptive statistics of the dataset

The variety and depth of the data used to train a model are crucial to its predictive power. We used a dataset with 192 experimental data points covering compressive strength, split tensile strength, and flexural strength to construct machine learning (ML) models in our study. By incorporating five crucial input parameters—cement, polycarbonate waste, water-to-cement ratio (w/c), slump, and water absorption—that significantly impact the anticipated results, we utilized this dataset to construct LASSO regression (LR) and decision tree (DT) models. Table 3 shows a detailed breakdown of all the important statistics and distributions for these input parameters. There are two main types of descriptive statistics: measures of central tendency and measurements of variability. One can learn much about the spread of data points from measures of variability, such as range (the lowest and maximum values of the variables), standard deviation, and variance. The mean, median, skewness, and kurtosis are central tendency measures that reveal how symmetrical and central the dataset is.

**Table 3:** Chemical properties of the polycarbonate waste ashes.

Sl. no	Chemical constituents	Percentage by weight
1	Cao	63.8
2	SiO_2_	19.7
3	AL_2_O_3_	5.7
4	Fe_2_O_3_	2.8
5	SO_3_	2.2
6	MgO	1.6
7	Na_2_O	0.3
8	K_2_O	0.7

**Figure 5:**
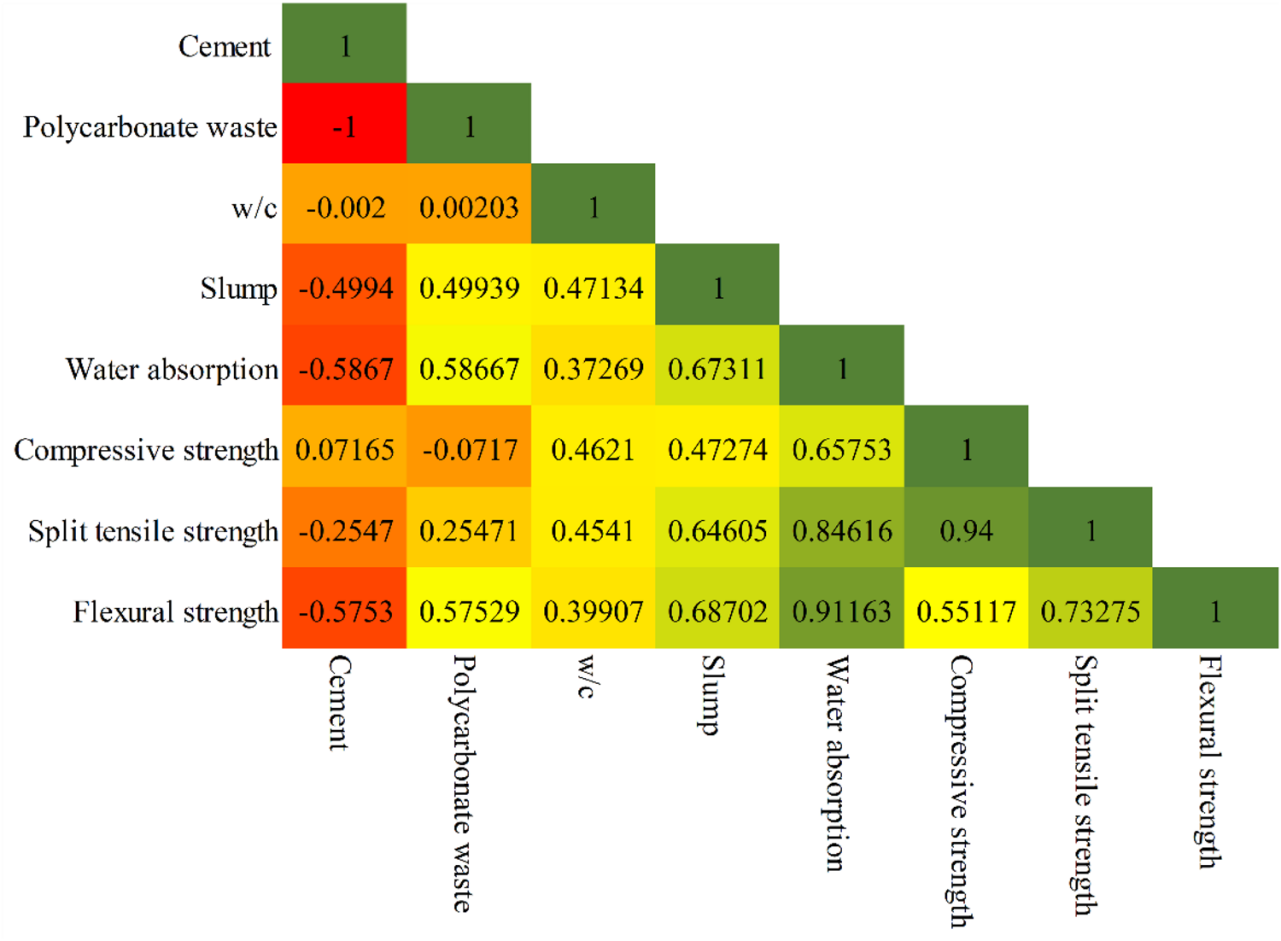
Pearson correlation matrix of the input and output parameters.

All five input parameters show very little deviation from a normal distribution, as shown in Table 4, which indicates that their skewness and kurtosis values are minimal as well. Because of this statistical feature, our dataset is more stable and trustworthy. We utilized the Pearson correlation coefficient to evaluate the level of correlation between the input and output parameters. Equation (2) defines the formula for the Pearson correlation coefficient R, where N is the sample size and ${input}_{i}$ and ${output}_{i}$ are the individual samples i. The average values of the samples are called ${\overline{input} }_{AV}$ and ${\overline{output} }_{AV}$. Only the linear relationship between two variables can be measured by the Pearson correlation coefficient, which has a value between − 1.0 and 1.0. The bottom three rows of the Pearson correlation matrix display the values of the Pearson correlation coefficient between each input and output variable (compressive strength, split tensile strength, flexural strength) (see Fig. 5). R’s proximity to or equality with 0 does not prove that two variables are independent.

2 $$R = \frac{{\mathop \sum \nolimits_{i = 1}^{N} \left( {input_{i} - \overline{input}_{AV} } \right)\left( {output_{i} - \overline{output}_{AV} } \right)}}{{\sqrt {\mathop \sum \nolimits_{i = 1}^{N} (input_{i} - \overline{input}_{AV} )^{2} } \sqrt {\mathop \sum \nolimits_{i = 1}^{N} (output_{i} - \overline{output}_{AV} )^{2} } }}$$

**Table 4:** Descriptive statistics of the input and output parameters.

	Unit	Mean	Median	Standard Deviation	Minimum	Maximum	Skewness	Kurtosis
Cement	kg/m3	306	306	55.5585	212	400	0	− 1.2010
Polycarbonate waste	kg/m3	94	94	55.5585	0	188	0	− 1.2010
w/c	–	0.42	0.43	0.06	0.3500	0.50	0.00	− 1.36
Slump	mm	48.973	49.256	8.8673	24.8889	62.2222	− 0.2789	− 0.5492
Water absorption	%	3.7620	3.6846	0.7402	2.3956	5.7294	0.4783	− 0.2801
Compressive strength	MPa	31.108	30.6276	5.1254	22.6722	49.0960	0.7146	0.8329
Split tensile strength	MPa	3.1733	3.1527	0.5332	2.2633	4.7212	0.4757	− 0.1489
Flexural strength	MPa	3.7763	3.6949	0.7206	2.5172	5.7294	0.5384	− 0.1739

Notable correlations are shown in Fig. 4, which displays the results of this analysis. There is a negative correlation (− 0.0717) for PC waste but strong positive correlations (0.4621), slump (0.47274), and water absorption (0.65503) for compressive strength. The results also show that there is a positive relationship between the amount of PC waste and the flexural and split tensile strengths. Insights into the factors impacting concrete strength characteristics are provided by these findings, which highlight the complex relationships between input parameters and output predictions. Statistical analysis and descriptive analysis revealed that the gathered database contained a wide range of experimental data. Thus, the availability of diverse data—as indicated by the correlation matrix and descriptive analysis—proves that each of the five inputs can be utilized as an input parameter to achieve the intended result.

## Results and discussion

### Workability of concrete

It is evident from the table that the workability of the concrete decreased with increasing replacement level. This may be because it is lighter than cement. As polycarbonate waste ash resides in a greater volume than cement, more water is required for lubrication. This results in a decrease in workability. Workability increases as cement is replaced by polycarbonate waste ash and is almost as good as that of conventional concrete when the cement is replaced by polycarbonate waste ash. Table 5 displays the workability of concrete and concrete made with PC waste ash as a fractional alteration of cement.

**Table 5:** Mix types of concrete and slump test.

Sl. no	Mix type	Slump in mm	Standard deviation
1	Conventional concrete	58	9.90
2	5% Polycarbonate waste ash	32	
3	10% Polycarbonate waste ash	43	
4	15% Polycarbonate waste ash	49	
5	20% Polycarbonate waste ash	56	
6	25% Polycarbonate waste ash	55	

### Density of concrete

The concrete density is a measure of its solidity. It is possible to modify the mixing process of concrete to produce a higher or lower density concrete product. The normal density of the concrete is 2400 kg/m^3^. The unit weight and density of concrete vary according to its aggregate content, air entrapment, water, and cement content. It is apparent from the table that the workability of the concrete decreases as the replacement level increases. The density did not change significantly; however, only a slight change was observed. A marginal decrease in the density of the PC waste ash when 10% was replaced correlated with the lower specific gravity of the cement when 10% was replaced^31^. The formation of more porous transition zones around ash particles likely contributed further to the density reduction. The densities of the control concrete and concrete containing PC waste ash as a partial replacement for cement are shown in Table 6. Concrete made from polycarbonate waste ash has a density comparable to that of conventional concrete, which is 2400 kg/m^3^.

**Table 6:** Mix types and density properties of the concrete in the compressive and split strength tests.

Mix type	Concrete density for compressive strength in kg/m^3^			Concrete density for split tensile strength in kg/m^3^		
	7 days	14 days	28 days	7 days	14 days	28 days
Conventional concrete	2426.48	2480.80	2453.15	2307.91	2294.71	2409.79
5% Polycarbonate waste ash	2416.61	2456.11	2371.18	2356.96	2334.32	2372.06
10% Polycarbonate waste ash	2398.83	2375.13	2390.93	2311.69	2339.99	2298.48
15% Polycarbonate waste ash	2408.71	2428.46	2414.64	2328.66	2306.03	2313.58
20% Polycarbonate waste ash	2410.84	2430.59	2416.77	2345.62	2340.12	2315.71
25% Polycarbonate waste ash	2415.25	2413.65	2422.31	2329.56	2306.93	2322.52

### Compressive strength

The compressive strength of a solid material is defined as its ability to sustain a maximum amount of compression under a progressive load without fracture. The compressive strength of a specimen is determined by dividing its maximum load by its cross-sectional area during a compression test. The compressive strength of the concrete specimens improved with replacement levels up to 20%, reaching a maximum strength at 20% and showing nearly the same strength when replaced with 5% replacement concrete. The improved packing density and accelerated hydration are the reasons for the strength enhancement at 20% replacement.

**Figure 6:**
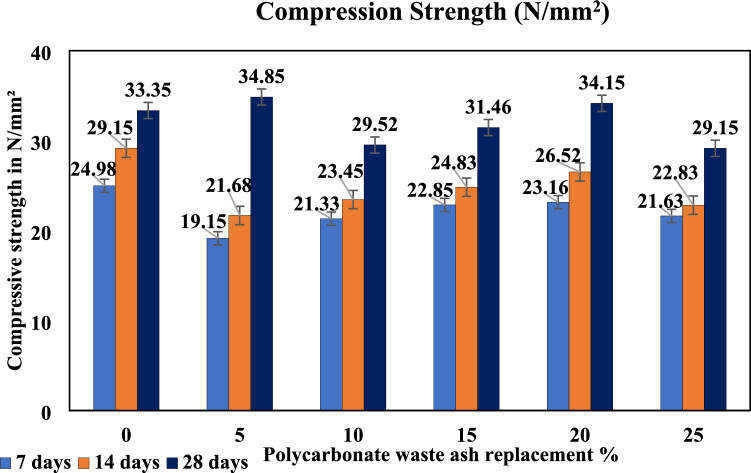
Compressive strength of the PC waste ash concrete mixtures.

The compression test is the most commonly performed test for hardened cement composites because of its ease of performance and because most of the desirable characteristics of concrete are qualitatively associated with its compressive strength. Compressive tests are conducted on cubical or cylindrical specimens. A 100 mm × 100 mm × 100 mm cube specimen was used in this experiment according to the standard. When PC waste ash is added to concrete, the compressive strength of the concrete decreases compared to that of conventional concrete. The compressive strength of the PC waste ash was good when it was replaced with 5% and 20%, but the compressive strength decreased when it was replaced with 25%. The reduction in density of the PC waste ash concrete may be responsible for this effect, as shown in Fig. 6.

### Split tensile strength

**Figure 7:**
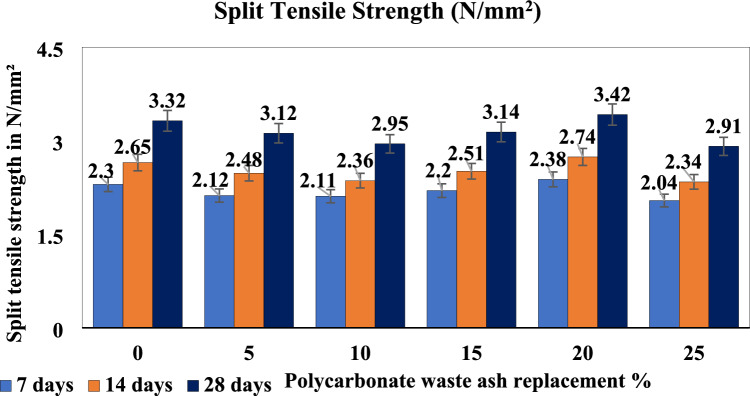
Split tensile strength of the polycarbonate waste concrete mixtures.

A cylindrical specimen is placed horizontally between the loading surfaces of a compression test machine and tested until it fails along its vertical diameter. Split tensile tests are performed on cylindrical specimens. This specimen has a diameter of 100 mm and a length of 150 mm. Figure 7 shows that the split tensile strength of biomedical waste ash concrete was found to be almost as high as that of conventional concrete. When 20% of the biomedical waste was replaced, the split tensile strength was excellent, while the 25% replacement strength was reduced compared with those of the 5% and 10% replacement strengths.

### Flexural strength

**Figure 8:**
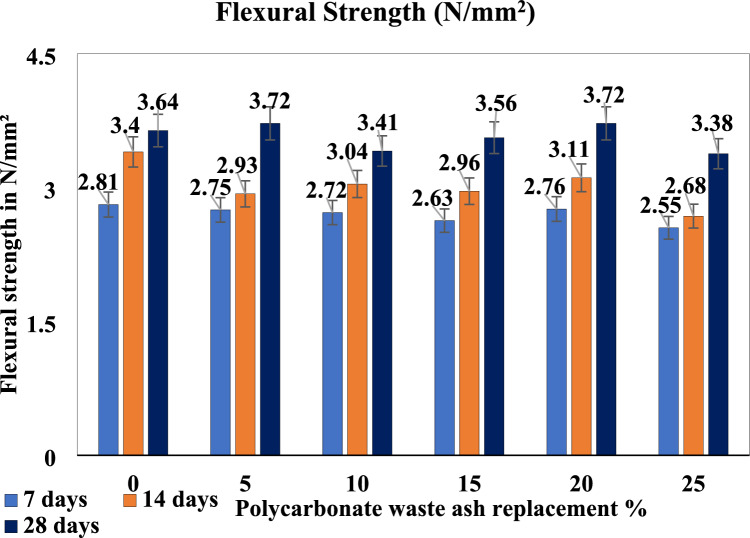
Flexural strength of the polycarbonate waste concrete mixes.

A flexural strength test was conducted on prismatic specimens with dimensions of 100 mm × 100 mm × 500 mm using a manual flexural strength testing machine. Tests were conducted on the beams at 7, 14, and 28 days after they had been cured in fresh water at 27 °C. The variation in flexural strength with different percentages of cement replacement by different admixtures. Figure 8 shows the flexural strength for different levels of cement replacement.

When considering polycarbonate in concrete, the differences in trends between flexural strength, splitting tensile strength, and compressive strength are influenced by the distinctive properties of polycarbonate and the methods of evaluation. Flexural strength assesses the material’s ability to withstand bending forces, which could be particularly relevant for polycarbonate due to its flexibility compared to traditional concrete components. The splitting tensile strength evaluates the resistance to forces applied perpendicular to the axis of the material, which may be significant for understanding how polycarbonate interacts within the concrete matrix. The compressive strength, which measures the resistance to inwards forces, is vital for assessing the overall stability and load-bearing capacity of concrete containing polycarbonate. The unique composition and structural behavior of polycarbonate within the concrete mix, along with factors such as aggregate size, distribution, and curing conditions, contribute to the variations observed in these strength properties.

### Durability tests

#### Water absorption test

**Figure 9:**
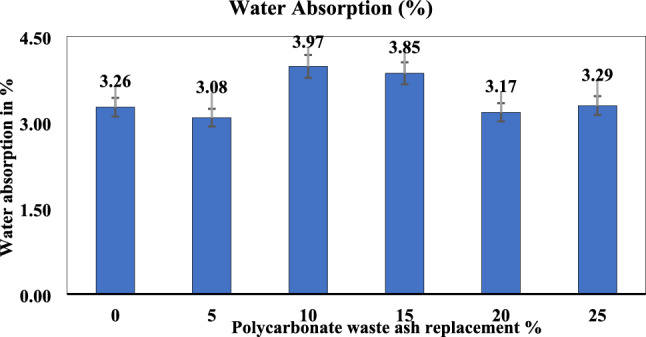
Water absorption test results for the PC waste concrete cubes.

A mortar cube was immersed in water for 28 days after casting for curing. The specimens were then oven-dried at 85 °C for 24 h until the mass became constant, after which they were weighed again. The weight was recorded as the dry weight of the specimen. A 24 h immersion in water at 85 °C was then performed on the specimen. This weight was then determined to be the dry weight of the specimen. When comparing standard concrete and polycarbonate concrete, the average difference in percentage weight was 21%. The 20% polycarbonate waste ratio provided a better water absorption index; however, the other ratios were comparatively higher, as shown in Fig. 9. Reduced water absorption is attributed to pore refinement by ash fillers and improved interfacial transition zones, as noted from SEM/EDX analysis. The greater water absorption observed in the 15% ash mixture than in the 0% and 5% ash mixtures could be attributed to a combination of these factors, including changes in porosity, chemical composition, void structure, and ash quality.

#### Acid attack test

**Figure 10:**
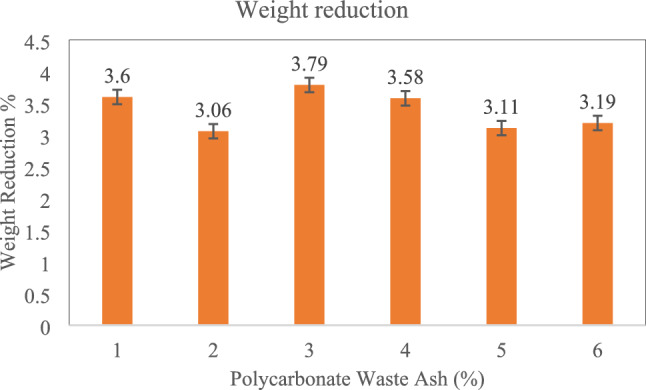
Acid curing test results for the PC waste concrete cubes.

The acid attack test involved the preparation of 150 mm × 150 mm × 150 mm concrete cubes with varying amounts of PC waste. A mould is used to cast and cure the specimens for a period of twenty-four hours. After twenty-four hours, the specimens were demolded and stored in a curing tank for twenty-eight days. After 28 days, all specimens were weighed and then immersed in a solution of 5% sulfuric acid (H2SO4) for 60 days. The 0.3 was the pH value of the acidic medium. Periodically, the pH of the acid solution was monitored and adjusted to maintain a constant level of 0.3. After the 60 days immersion period, the specimens were carefully extracted, rinsed with running water, and left to air-dry for 2 days to ensure consistent weight. Next, the specimens were weighed to determine the extent of weight loss, enabling the calculation of percentage loss, as depicted in Fig. 10. Acid resistance is attributed to decreased calcium content and densification within the specimens, factors crucial for maintaining structural integrity in aggressive environments^53^.

### Microstructural properties

#### SEM and EDX analysis

**Figure 11:**
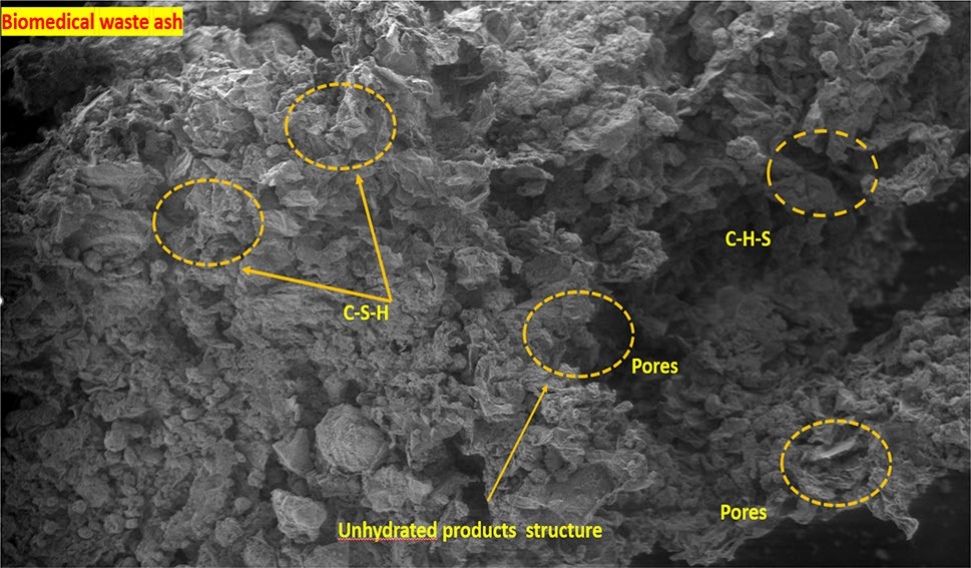
SEM image of 20% polycarbonate waste ash concrete.

Figure 11 shows an SEM image of the PC waste ash. The cloudy white substance observed in the image is calcite. The presence of calcite is clearly observed in the image. The addition of calcite to the concrete improved the binding properties. A glassy-like substance is observed in the image because the material is amorphous with silicate present. This also proves that when polycarbonate waste is used in concrete, it can aid in the formation of C–S–H gel.

**Figure 12:**
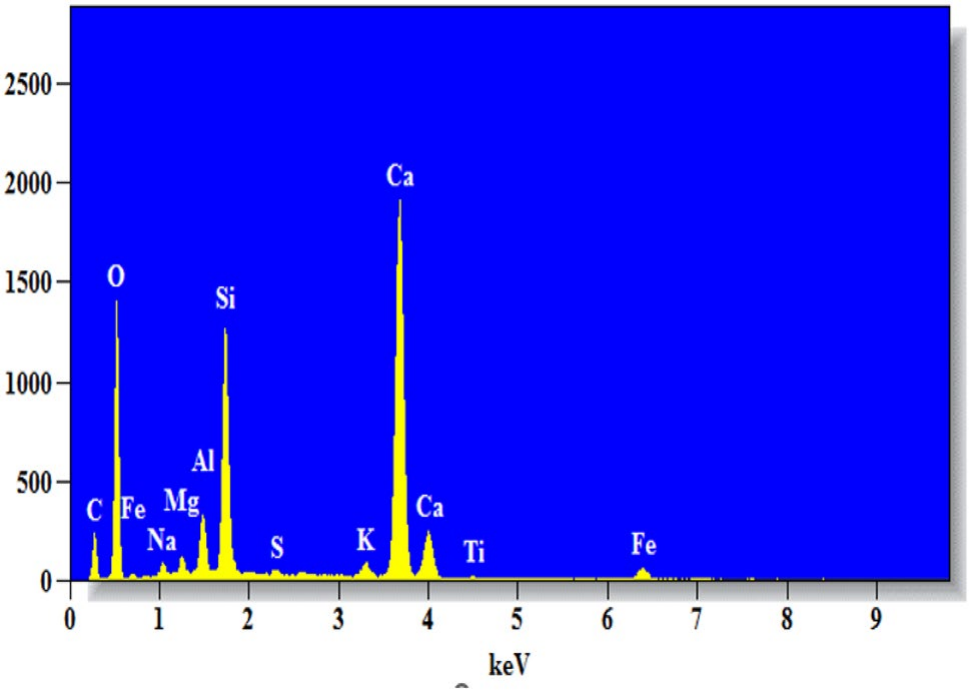
EDS for 20% polycarbonate waste ash concrete.

Figure 11 shows SEM images of the concrete mixtures used with biomedical waste ash. The results show the presence of various hydration products and the particle distribution. This was used for the analysis of the pores present in the concrete. The specimens of concrete tested at 28 days were used for SEM tests. The white spherical particles are C–S–H gels. There is also a small amount of unhydrated materials in the concrete mix. The small circular areas represent the pores that restrict the binding properties of the aggregates and mortar. Figure 12 shows the EDS analysis of the 20% biomedicine waste ash mixture. The major elements present are silicon, calcium, aluminum, and oxygen.

#### XRD analysis

From the XRD pattern of the biomedical waste ash, the major compound observed was quartz at 2θ = 26.677°, 20.899°, and 50.161°. Other compounds present in PC waste ash are lime, aluminum oxide and iron oxide. From the results, it is clear that the major compound present was silicon oxide.

**Figure 13:**
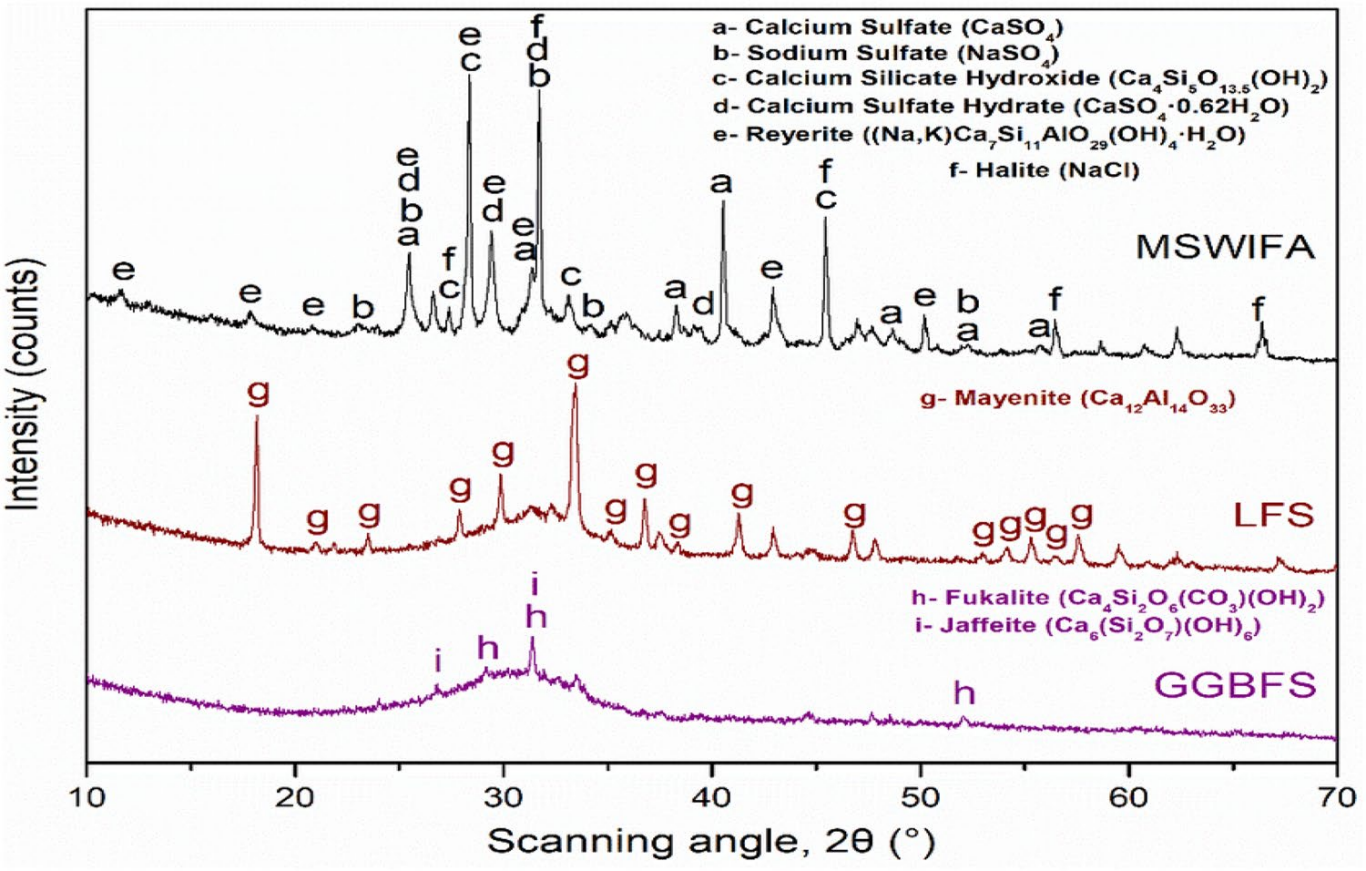
XRD pattern for 20% polycarbonate waste ash concrete.

Figure 13 shows the XRD pattern for a 20% polycarbonate waste ash mixture. Since many peaks are observed, it is said to be crystalline in nature. The major peak indicates quartz, corresponding to 2θ = 26.590 at plane (0 1 1), which matches [ICDD = 041447]. The second major peak identified was portlandite, corresponding to plane (1 0 1) at 2θ = 34.102°. Considering durability, portlandite forms a weak bond between mortar and aggregates, which is the main reason for the increase in porosity in concrete. The other minor compounds present are C–S–H, calcite, ettringite and kyanite.

### Cost analysis

The development of the construction sector is now increasing, but it needs to be economical. In building social change, the cost parameters of every project are taken. The prices for conventional concrete depend on the cost of the cement and construction materials, such as the addition of aggregates and water^20^. Material cost may also depend upon the transportation, locality available, and market demand. This project is shown in Table 7. The materials used were cement, coarse aggregate, river sand, and polycarbonate waste. The price of the cement was 420 Rs/bag, and one bag was 50 kg. Coarse aggregate was purchased at 1450 Rs/ton. River sand was purchased at 1100 Rs/ton. The polycarbonate waste ash processing charge was 5 Rs/kg, as explained in Table 7.

**Table 7:** Cost analysis for standard and 20% polycarbonate waste ash concrete mixes.

S. no	Materials	Rate (Rs)	Unit	Conventional concrete		20% Polycarbonate waste ash	
				Quantity in kg	Amount in Rs	Quantity in kg	Amount in Rs
1	Cement	420	50 kg	390	3276	312	2620
2	Coarse aggregates	1450	MT	1180	1829	1180	1829
3	Fine aggregates	1100	MT	765	918	765	918
4	Polycarbonate waste	5	kg	–	–	78	390
Total				6023		5757	

### ML results

#### Model training and validation

To create regression models, the dataset is divided into training and testing sets. The proportion of the training set to the testing set is 70:30, which indicates that 70% of the data are used to train the model and that the remaining 30% are used to evaluate it. Trial-and-error tuning was employed in the training phase to ascertain the hyperparameters, the models’ structures, and their functions. The current study predicts three outputs—compressive strength (MPa), split tensile strength (MPa), and flexural strength (MPa)—using two cutting-edge, state-of-the-art machine learning algorithms. Table 8 summarizes the ideal decision tree (DT) and LASSO regression (LR) hyperparameters and architectures for the three outputs.

**Table 8:** Optimized hyperparameters of the LR and DT models.

	Hyperparameter	Optimized value		
		Compressive strength (MPa)	Split tensile strength (MPa)	Flexural strength (MPa)
LR	max_iteration	1000	900	900
	alpha	0.8	0.5	1
DT	max_depth	16	14	16
	min_samples_split	4	3	2
	min_samples_leaf	2	2	3

#### Statistical analysis of the results

Table 9 displays five commonly used performance metrics that are used to evaluate the accuracy and reliability of the predictive models. Table 9 displays the mathematical expression along with its ideal value.

**Table 9:** Performance metrics for accuracy evaluation.

Performance metrics	Mathematical expression	Ideal value
Coefficient of determination (R^2^)	$R^{2} = \frac{{\mathop \sum \nolimits_{i = 1}^{n} \left( {actual - actual_{avg} } \right)^{2} - \mathop \sum \nolimits_{i = 1}^{n} \left( {actual - predicted} \right)^{2} }}{{\mathop \sum \nolimits_{i = 1}^{n} \left( {actual - actual_{avg} } \right)^{2} }}$	Should be close to 1 (Ideal = 1)
Root mean square error (RMSE)	$RMSE=\sqrt{\frac{1}{n}\sum_{i=1}^{n}{(actual-predicted)}^{2}}$	Should be close to 0 (Ideal = 0)
Mean absolute error (MAE)	$MAE=\frac{1}{n}\sum_{i=1}^{n}\left|(predicted-actual)\right|$	Should be close to 0 (Ideal = 0)
Variance account factor (VAF)	$VAF = \left( {1 - \frac{{var\left( {actual - predicted} \right)}}{{var\left( {actual} \right)}}} \right) \times 100$	Should be close to 100 (Ideal = 100)
Nash–Sutcliffe efficiency (NS)	$NS=1-\frac{{\sum }_{i=1}^{n}{(actual-predicted)}^{2}}{{\sum }_{i=1}^{n}{(actual-{actual}_{avg})}^{2}}$	Should be close to 1 (Ideal = 1)

Tables 10 and 11 provide the R^2^, RMSE, MAE, VAF, and NS of the predictions for the training and testing datasets, respectively. This study emphasizes the intelligent presentation of performance metrics using unnormalized data. The reader is given a clear and direct grasp of complex results by departing from typical normalizing procedures. The research discourse is enriched by this departure because it promotes conceptual clarity and provides an unobstructed perspective of data patterns. For the output compressive strength, the R^2^ values were 0.830916 and 0.97691 for LR and DT, respectively, in the training set, whereas the R^2^ values in the testing set were 0.725038 for LR and 0.853683 for the DT model. This indicates the strong learning and generalization abilities of the DT model in comparison to those of the LR model. The R^2^ values for the LR and DT models for the output split tensile strength in the training set were 0.908856 and 0.984817, respectively, which are significantly better than the R^2^ values for compressive strength predictions (Table 10). For flexural strength predictions, R^2^ values in training and testing were greater for both the LR and DT models; thus, both models offer better predictions than the other two models. In the testing stage, the R^2^ and RMSE values for the DT model were 0.91197 and 0.186847, respectively, whereas those for the LR model were 0.909 and 0.245354, respectively. The RMSE and MAE values of the DT models for all three outputs, compressive strength, split tensile strength, and flexural strength, are significantly lower in both the training and testing stages than those of the LR model, as shown in Tables 10 and 11. The results thus demonstrate that the DT model yields better predictions than the LR model. The values of additional performance indicators of the models are shown in Tables 10 and 11.

**Table 10:** Performance parameter values for the training phase.

	Training				
	R^2^	RMSE	MAE	VAF	NS
Compressive strength					
LR	0.830916	2.144854	1.559202	83.05	0.811463
DT	0.97691	0.775969	0.357985	97.53	0.975323
Split tensile strength					
LR	0.908856	0.225757	0.187434	90.83	0.823317
DT	0.984817	0.069161	0.036175	98.34	0.983418
Flexural strength					
LR	0.979964	0.151603	0.10935	97.82	0.960484
DT	0.997059	0.042727	0.028122	99.68	0.996861

**Table 11:** Values of the performance parameters for the testing phase.

	Testing				
	R^2^	RMSE	MAE	VAF	NS
Compressive strength					
LR	0.725038	3.017904	2.031111	72.31897	0.690061
DT	0.853683	2.094364	1.107639	85.09227	0.850731
Split tensile strength					
LR	0.764699	0.277734	0.221383	76.31102	0.710915
DT	0.879403	0.179651	0.109423	87.93709	0.879045
Flexural strength					
LR	0.909	0.245354	0.166399	90.23678	0.833787
DT	0.91197	0.186847	0.09974	91.1873	0.903606

#### Scatter plots

**Figure 14:**
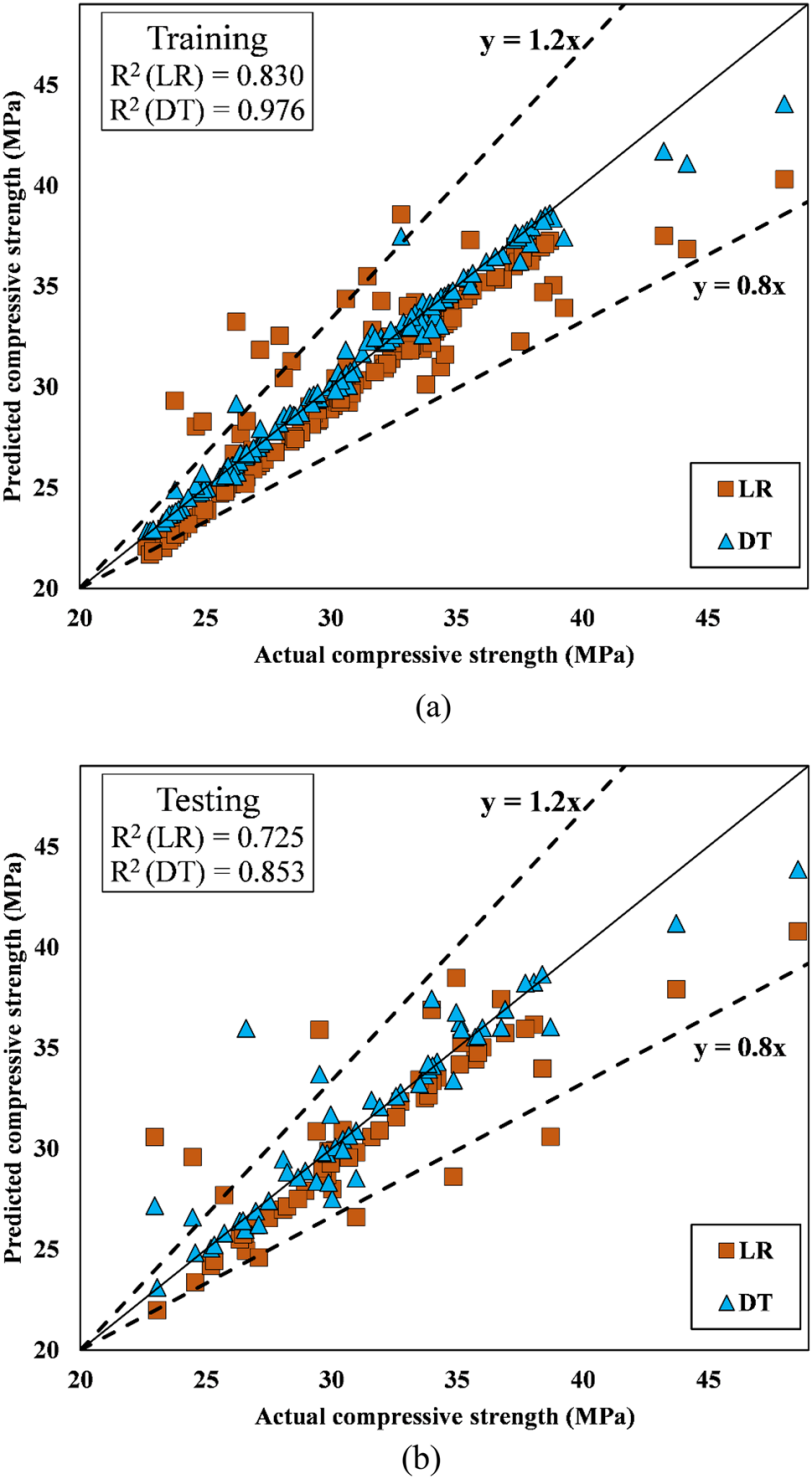
(**a**) Scatter plot of the compressive strength during training. (**b**) Scatter plot of the compressive strength during testing.

**Figure 15:**
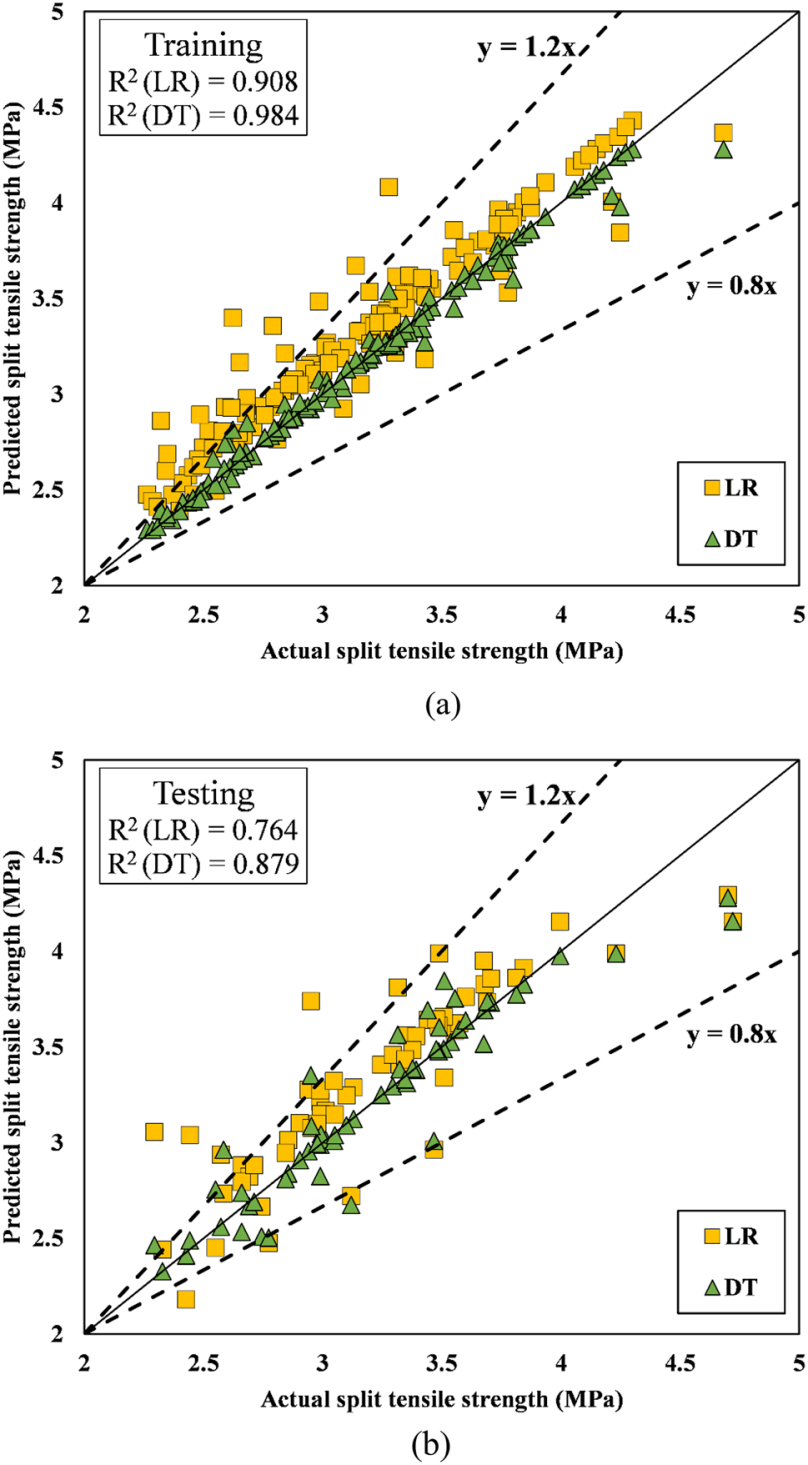
(**a**) Scatter plot of the split tensile strength during training. (**b**) Scatter plot of the split tensile strength during testing.

**Figure 16:**
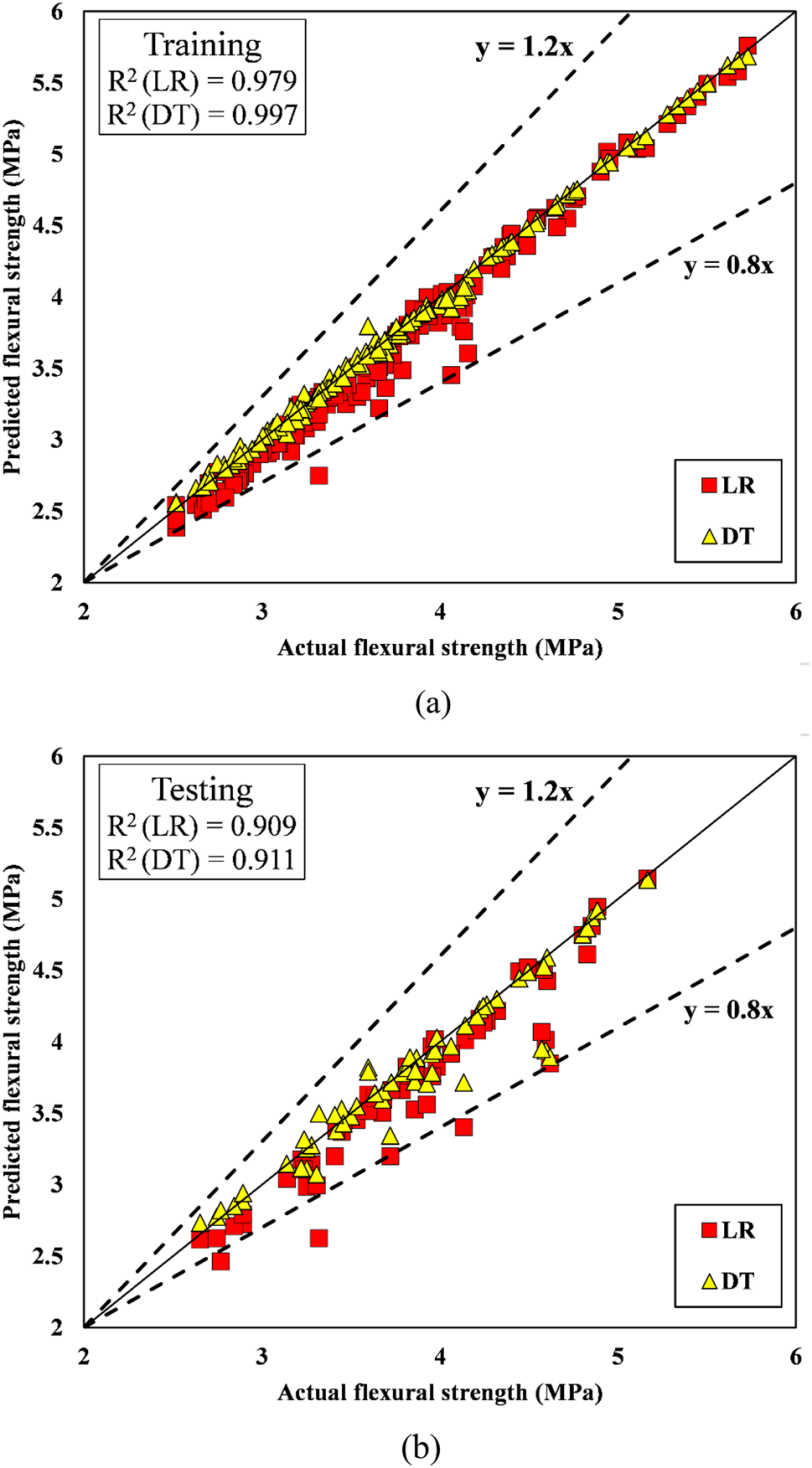
(**a**) Scatter plot of the flexural strength during training. (**b**) Scatter plot of the flexural strength during testing.

The scatter plots for the training and testing stages of the compressive strength prediction models (LR and DT, respectively) are shown in Figs. 14a,b, respectively. Placing the data point on the line (y = x) will result in the best-performing model. Figure 13a shows that during the training phase, the predicted and observed values of compressive strength for the DT models lie exactly on the line (y = x), but for LR, the data points were found to be off kilter. Additionally, compared to those in the training stage, the DT data points in the testing stage are slightly off-kilter with respect to the line (y = x). The regression curve comparing the actual and predicted values for each data point for the DT and LR models in the training and testing stages is shown in Fig. 15a,b for the split tensile strength. During training, the predictions of the DT model are closer to the actual values of the split tensile strength than those of the LR model. Figure 15b shows that the DT model performs poorly in the testing stage compared to its performance in the training stage, and it fails miserably to maintain its performance. Figure 16a, b display the scatter plots for flexural strength prediction using the DT and LR models. It is evident that the data points in both the training and testing stages are much closer to the reference point than are the points predicted for the split tensile strength and compressive strength.

#### REC curve

**Figure 17:**
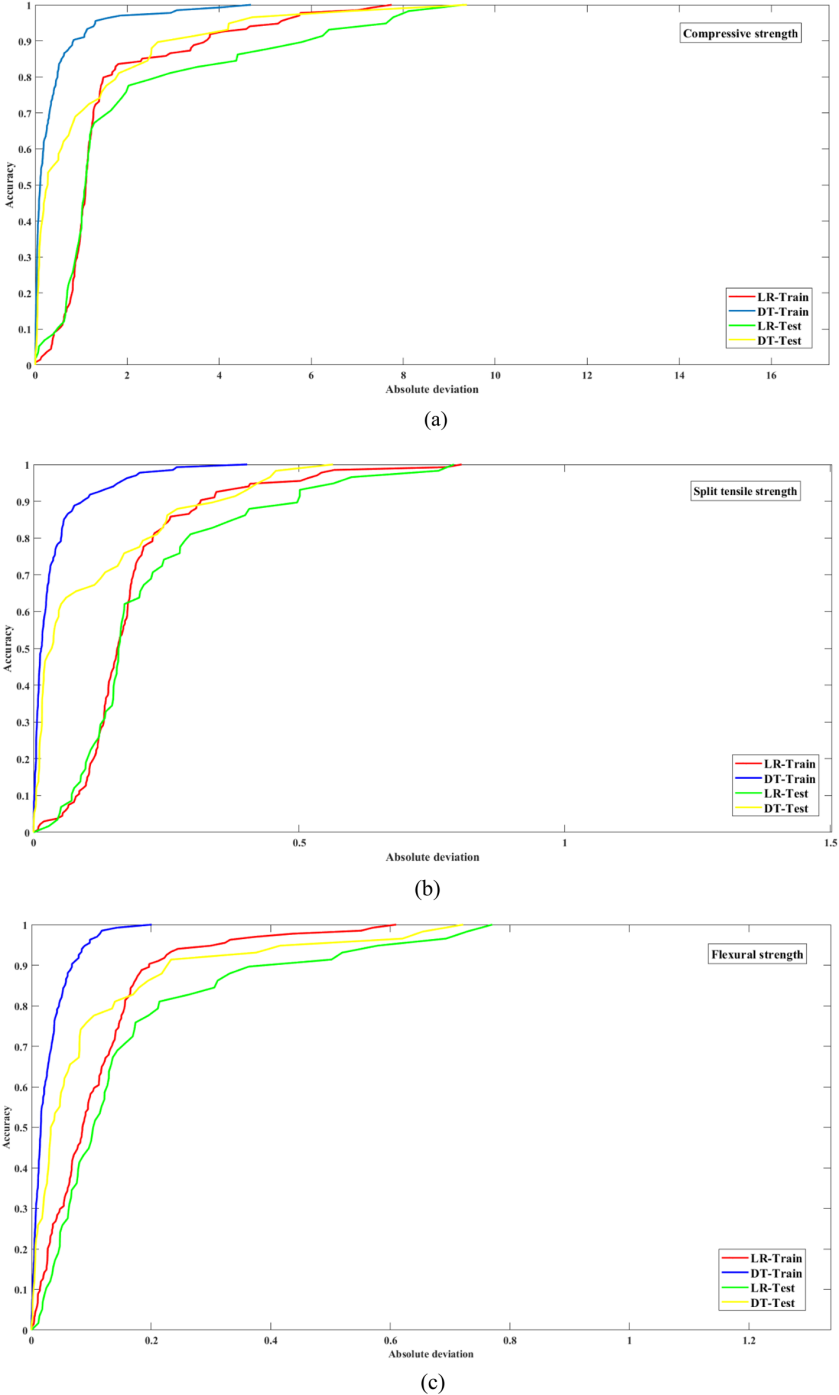
(**a**) REC curve of the compressive strength during training and testing. (**b**) REC curve of the split tensile strength during training and testing. (**c**) REC curve of the flexural strength during training and testing.

An effective tool for evaluating and comparing multiple regression models at once in a single graphical representation is the regression error characteristic (REC) curve^54–56^. Basically, it is a great tool for comparing how various regression functions work. It is easy to compare different regression models side by side on the REC graph because each monotonically increasing curve represents a different model. The x-axis shows the margin of error for the regression function, while the y-axis shows its accuracy. More information about the modelling process can be gleaned from the curve’s shape. It is believed that models with a closer placement to the top left corner of the graph are more accurate. In addition, the AOC represents the regression model’s expected error. With more faith in the model’s predictions and a smaller AOC value, we can say that the model is more accurate overall. The REC graphs for the training and testing phases of the LR and DT models are shown in Fig. 17a–c. These graphs focus on output parameters such as the flexural strength, compressive strength, and split tensile strength. Visually examining these graphs makes it easy to visualize patterns. Figure 17a,b show that the LR model predicts compressive and split tensile strengths with relatively lower accuracy during testing and training. However, in regard to predicting flexural strength, both the LR and DT models show strong accuracy during training (Fig. 17c). However, in regard to testing time, both models start to fall short, and the REC curves start to stray far from the top left. The AOC values for the three output parameters are shown in Table 12, which provides further information. For all three outputs, the DT model outperforms the LR model in terms of the AOC during both training and testing. Overall, the DT model outperforms its LR counterparts in the REC analysis, especially in regard to predictive accuracy and robustness throughout the different stages of model evaluation.

**Table 12:** AOC values in training and testing.

Tests	Models	AOC	
		Training	Testing
Compressive strength	LR	1.5303	1.9508
	DT	0.3405	1.0267
Split tensile strength	LR	0.1844	0.2146
	DT	0.0347	0.1046
	DT	0.0274	0.0935

#### SHAP interpretations

**Figure 18:**
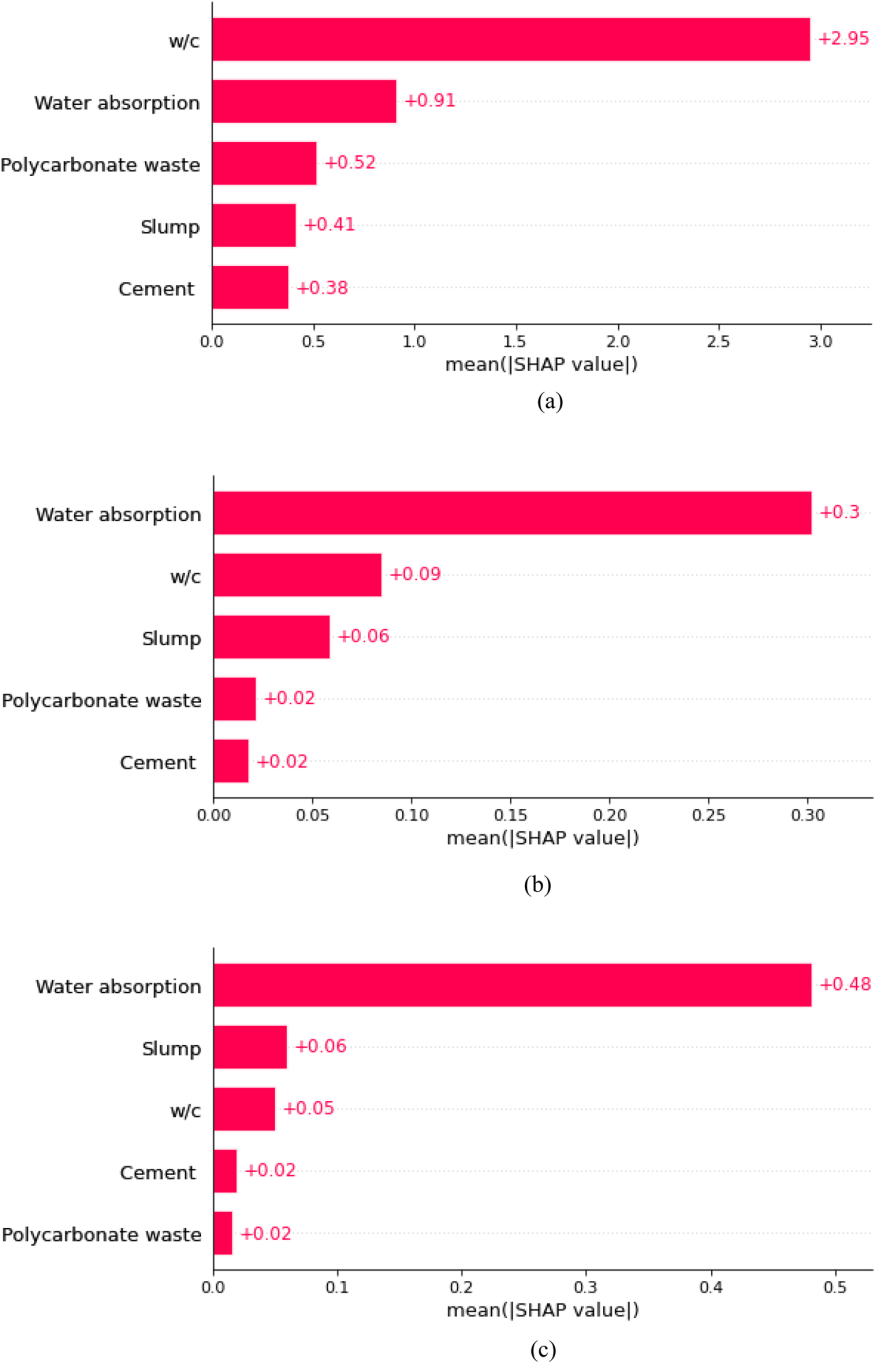
(**a**) Mean SHAP values for compressive strength. (**b**) Mean SHAP values for the split tensile strength. (**c**) Mean SHAP values for the flexural strength.

Delicately untangling the complex web of interactions and connections among the myriad features it considers, this study dives deep into the intricate workings of a decision tree (DT) model. Using Shapley values derived from coalition-based game theory, the relationship between features and specific predictions is precisely explained through an insightful exploration using SHapley Additive exPlanations (SHAP) analysis^57–59^. Shapley values provide a detailed depiction of the impact of each feature by painstakingly averaging all possible combinations of feature values. The final, all-encompassing global feature influence values are obtained by averaging these Shapley values for every feature in the dataset. Then, to make the feature importance clear, these values are presented in an attractive way, with a descending order of importance. Figures 18a–c show the DT model’s mean SHAP values, which explain the predictions for different parameters, such as compressive strength, split tensile strength, and flexural strength. Figure 18a shows that the top three variables for compressive strength prediction are water absorption, w/c (water-to-cement ratio), and PC waste. These three variables have the highest mean SHAP values: 2.95, 0.91, and 0.52, respectively. Water absorption, w/c, and slump are the three most important factors for predicting the split tensile strength (Fig. 18b), with average SHAP values of 0.3, 0.09, and 0.06, respectively. Figure 18c shows that water absorption is the most important factor in predicting flexure strength, with slump and w/c following closely behind with SHAP values of 0.48, 0.06, and 0.05, respectively. Because these results depend on the dataset used in this study, it is critical to note that they are contextual. Nevertheless, there is hope for the future of this dataset and its potential to improve these results, leading to more precise and accurate analyses.

## Future perspective

To formulate mix design procedures, researchers have conducted different trials with polycarbonate waste ash. Therefore, a sophisticated mix design process is necessary because several variables, such as cement and polycarbonate waste ash binders, must be considered. Although mix design procedures vary according to the material sources, the mix design technique can be applied. It is vital that a mixed design procedure be developed for polycarbonate waste ash concrete. In terms of structural elements, polycarbonate waste ash concrete offers improved structural properties and durability.

Many studies have been conducted on the durability of polycarbonate waste ash concrete in the short term, but very few have been conducted in the long term. The long-term study of PC waste ash concrete might prove beneficial for applications in commercial and industrial settings. The theory and understanding of conventional mixture proportions can be easily applied to the construction of polycarbonate waste ash mixtures with comparable results in terms of workability and strength for aggregate grading, strength, and angularity. Recommendations are made for the formulation of mixture proportions and the use of superplasticizers to improve workability and compatibility. It has been demonstrated that the tensile strength of polycarbonate waste ash concrete is superior, and research into the shear strength of geopolymer concrete in beam applications is suggested and is currently being conducted to fully exploit this property of polycarbonate waste ash concrete. Additionally, life cycle assessment (LCA) and environmental impact analysis of polycarbonate waste ash concrete compared to conventional concrete are important areas for future study.

## Conclusions

Researchers have investigated the feasibility of using polycarbonate waste ash as a filler in M25 grade concrete. The conclusions drawn from the present work are as follows:

The density of the concrete decreases slightly as the replacement level of the PC waste ash increases. A replacement of up to 20% is effective. The workability of polycarbonate waste ash-based concrete is inferior to that of traditional concrete. Polycarbonate waste ash concrete (PCWAC) with 5% to 20% replacement shows greater compressive strength than traditional concrete. However, at 25% replacement, the strength decreases compared to that at 20% replacement. The split tensile and flexural strengths of PCWAC are similar to those of conventional concrete. Based on the research results, the optimal replacement level is determined to be 20%. PCWAC exhibits lower water absorption than traditional concrete. Compared with standard concrete, PCWAC shows less weight loss in acid resistance tests. The quantities of reactive silica and alumina in various polycarbonate wastes play a crucial role in determining the overall performance of the final product. The utilization of PC waste in concrete contributes to the development of new materials, pollution reduction, and economic improvement through the circular economy.

In this innovative study, we also developed predictive models for the compressive, split tensile, and flexural strengths of concrete using least absolute shrinkage and selection operator (LASSO) regression (LR) and decision tree (DT) methods. The complex dynamics of concrete behaviour were captured by two models constructed from a wide variety of concrete mix components, such as cement, polycarbonate waste, w/c, slump, and water absorption. We optimized each model’s hyperparameters to achieve peak performance through extensive experimentation and careful parameter tuning. We painstakingly evaluated the performance of our models using a suite of commonly used metrics, including R^2^, RMSE, MAE, VAF, and NS. The DT model demonstrated unmatched predictive accuracy across key metrics, making it the clear winner in the testing crucible. Its exceptional performance in predicting compressive strength (R^2^ = 0.853683, RMSE = 2.094364), split tensile strength (R^2^ = 0.879403, RMSE = 0.179651), and flexural strength (R^2^ = 0.91197, RMSE = 0.186847) solidified its status as a go-to option for predicting these strength parameters. Using SHAP analysis, we also investigated the complex relationship between the input parameters and the output predictions. This advanced method revealed important characteristics that significantly impacted individual predictions, providing invaluable information about the processes that shape concrete performance.

## Data Availability

The data have been reported in the manuscript.
